# Detection and Genetic Characteristics of H9N2 Avian Influenza Viruses from Live Poultry Markets in Hunan Province, China

**DOI:** 10.1371/journal.pone.0142584

**Published:** 2015-11-10

**Authors:** Yiwei Huang, Hong Zhang, Xiaodan Li, Shixiong Hu, Liang Cai, Qianlai Sun, Wenchao Li, Zhihong Deng, Xingyu Xiang, Hengjiao Zhang, Fangcai Li, Lidong Gao

**Affiliations:** 1 Hunan Provincial Center for Disease Control and Prevention, Changsha, Hunan, People’s Republic of China; 2 National Institute for Viral Disease Control and Prevention, Chinese Center for Disease Control and Prevention, Beijing, People’s Republic of China; University of Edinburgh, UNITED KINGDOM

## Abstract

H9N2 avian influenza viruses (AIVs) are highly prevalent and of low pathogenicity in domestic poultry. These viruses show a high genetic compatibility with other subtypes of AIVs and have been involved in the genesis of H5N1, H7N9 and H10N8 viruses causing severe infection in humans. The first case of human infection with H9N2 viruses in Hunan province of China have been confirmed in November 2013 and identified that H9N2 viruses from live poultry markets (LPMs) near the patient’s house could be the source of infection. However, the prevalence, distribution and genetic characteristics of H9N2 viruses in LPMs all over the province are not clear. We collected and tested 3943 environmental samples from 380 LPMs covering all 122 counties/districts of Hunan province from February to April, 2014. A total of 618 (15.7%) samples were H9 subtype positive and 200 (52.6%) markets in 98 (80.3%) counties/districts were contaminated with H9 subtype AIVs. We sequenced the entire coding sequences of the genomes of eleven H9N2 isolates from environmental samples. Phylogenetic analysis showed that the gene sequences of the H9N2 AIVs exhibited high homology (94.3%-100%). All eleven viruses were in a same branch in the phylogenetic trees and belonged to a same genotype. No gene reassortment had been found. Molecular analysis demonstrated that all the viruses had typical molecular characteristics of contemporary avian H9N2 influenza viruses. Continued surveillance of AIVs in LPMs is warranted for identification of further viral evolution and novel reassortants with pandemic potential.

## Introduction

H9N2 avian influenza viruses (AIVs) are highly prevalent in domestic poultry in Asia since the early 1990’s [1, 2]. These viruses are lowly virulent for poultry, frequently cause mild disease and rarely result in poultry deaths. However, they have been associated with severe morbidity and mortality in poultry when they co-infected with other pathogens [3]. Occasionally H9N2 viruses have been identified in pigs and humans [4–6]. H9N2 AIVs in Asia can be divided into three main lineages represented by their prototype strains based on antigenic and phylogenetic analysis of hemagglutinin: A/Chicken/Beijing/1/94 (BJ94) or the A/Duck/Hong Kong/Y280/97 (Y280), A/Quail/Hong Kong/G1/97 (G1), and A/Duck/Hong Kong/Y439/97 (Y439) or A/Chicken/Korea/96323/96 [7]. The BJ94-like viruses are primarily prevalent in chickens and have beengradually substituted by F98-like viruses represented by A/Ck/Shangha/F/98 (F98) in the East and South China [8]. According to the constellation of eight viral gene segments, multiple genotypes of H9N2 viruses have been generated by complicated gene segment reassortment [9]. At least 98 genotypes are divided into seven series (A-G) worldwide from 1966 to 2009 [9]. In addition, a novel genotype (G57) or genotype S represented by A/chicken/Zhejiang/HJ/2007(HJ07) emerged in 2007 and became predominant in farm chickens in China since 2010 [10, 11].

H9N2 viruses show a high genetic compatibility with other subtypes of AIVs and have been involved in the genesis of novel AIVs causing severe infection in humans [1, 12, 13]. Highly pathogenic avian influenza H5N1 viruses caused the first human infection in Hong Kong in 1997 [14]. High homology of virus genomes suggested that internal genes of some H9 viruses were closely related to the human H5N1 influenza viruses of Hong Kong [1, 15]. The novel H7N9 AIVs that have been firstly identified in Anhui province and Shanghai city of China have quickly spread to several provinces and led to many human infections and deaths since 2013. Gene sequence analysis of the pathogens showed that all six internal genes of the H7N9 viruses were derived from H9N2 viruses [16, 17]. A similar conclusion applies to the human-infecting H10N8 viruses in Jiangxi province of China in 2013 [13].

Circulation of H9N2 viruses in live poultry markets (LPMs) continues to pose a serious public health threat. Like other developing countries, LPMs in China are part of the everyday food supply chain that offer poultry meat and live birds for sale. However LPMs also provide sites for different subtypes of avian influenza virus mixing, transmission and exchange of gene segments, and for zoonotic transfer between humans and poultry [18, 19]. Thus, LPM exposure history is considered to be a main risk factor for human infection with AIVs [20, 21]. Closure of LPMs has a measurable effect on infection spread control [21, 22].

In Hunan province of China, influenza monitoring continues to catch human cases infected with AIVs. Six cases of human infection with H5N1 AIVs have been confirmed in the province including the first case in mainland China since 2005, while the first laboratory-confirmed case of human infection with H9N2 viruses in the province was diagnosed in November 2013 [23–25]. Epidemiological investigation identified that H9N2 viruses from LPMs near the patient’s house could be the source of infection [25]. However, the ecology of H9N2 viruses in LPMs all over the province is not clear. For a comprehensive assessment of the ecology of AIVs in LPMs in Hunan province, as well as providing a scientific basis for prediction and warning of possible influenza pandemics, here we describe the detection and genetic characteristics of H9N2 viruses in LPMs of Hunan province in 2014.

## Materials and Methods

### Sample collection

Hunan province lies in south-central China and is administratively divided into 14 cities, 122 counties/districts. Large-scale live poultry wholesale and retail markets of each county/district in Hunan province were selected for the study. At least one LPM was select for each sampling of each county/district based on the number of large-scale LPMs. Samples were collected twice a month for each county/district from February to April, 2014, which was thought to be the high risk season for human infection with avian influenza in Hunan province. If a county/district had only one large-scale LPM, then six samplings would be in this market. If a county/district had a number of markets, Sampling would rotate among different markets. This study was conducted in a total of 380 LPMs covering all 122 counties/districts of Hunan province (S1 Table). The range of birds sold at the markets included chickens, ducks, geese and pigeons.

Each selling stall in LPMs was selected as a sampling point. Generally, two to three sampling points were selected for each market. At least three environmental samples involving poultry drinking water, sewage, poultry feces, cage swabs and chopping board swabs were collected for each sampling at each sampling point, preferentially for poultry drinking water and sewage.

Samples were placed in 15ml tubes containing 3ml virus transport media which contained: DMEM (Thermo Scientific Hyclone, Logan, USA), 0.5% BSA (Sigma, St Louis, USA), 10% glycerol, 2x10^6^ U/L penicillin G, 200 mg/L streptomycin, 2x10^6^ U/L polymyxin B sulfate, 250mg/L gentamicin (Sigma, St Louis, USA). Samples were immediately transported to monitoring laboratories at 4°C, stored at −70°C, and tested within one week.

### Sample detection and subtyping of AIVs

Environmental samples were tested by real-time reverse transcription PCR assays for influenza A, and positive samples for influenza A were further tested for H9, H7 and H5 subtypes. Specifically, samples were mixed by vortexing at 3000 r/min for 15s and centrifuged at 3000r/min for 10min. RNA was extracted from the suspension using QIAamp Viral RNA Mini Kit (QIAGEN, Hilden, Germany) and tested using SuperScript III Platinum One-Step qRT-PCR kit (Invitrogen, Carlsbad, USA) on 7300 Real-Time PCR System (Applied Biosystems, Foster City, USA), in accordance with the manufacturer’s instructions. Sequences of primers and probes are based on WHO information for molecular diagnosis of influenza virus [26]. Samples with a cycle threshold (Ct) value of less than 35 were determined to be positive.

### Virus isolation and genome sequencing

Virus isolation and genome sequencing were conducted by a previous method [27]. Specifically, virus isolation was conducted from samples which tested positive for influenza H9 subtype using 10-day-old SPF embryonated chicken eggs based on the manual for the laboratory diagnosis and virological surveillance of influenza as recommended by the World Health Organization. Viral RNA was extracted from the harvested liquids using QIAamp Viral RNA Mini Kit (QIAGEN, Hilden, Germany). Entire coding sequences of the genomes were amplified using OneStep RT-PCR Kit (QIAGEN, Hilden, Germany). PCR products were sequenced using the Big Dye Terminator Cycle Sequencing Ready Reaction kit (Applied Biosystems, Foster City, USA) on Applied Biosystems 3730xl DNA analyzer (Applied Biosystems, Foster City, USA), following the manufacturer’s instructions.

### Phylogenetic analysis

The genome sequence alignments were performed using a built-in MUSCLE program in the MEGA software package version 6.06. Phylogenetic analysis was based on open reading frames of complete genome sequences of the eight segments. Sequences of the representative H9N2 lineages and H9N2 isolates of Hunan province were downloaded from GenBank and included in the phylogenetic analyses. Phylogenetic trees were constructed using neighbor-joining method by means of bootstrap analysis with 1000 replications in the MEGA software version 6.06, with the Tamura-Nei model. Genotypic analysis was classified according to genetic distances and topologies of phylogenetic trees. Generally, genes sharing over 95% homology in the same lineage were considered as a genotypic group.

### Statistical analysis

SPSS (version 13.0) was used for statistical analysis. Differences between proportions were analyzed using χ^2^ test and a difference was considered statistically significant when p<0.05.

### Nucleotide sequence accession numbers

The complete genome nucleotide sequences of eleven H9N2 viruses generated in our study were submitted to the GenBank database and the accession numbers are KT356718 to KT356805.

### Ethics statement

This study was approved by the institutional review board of Hunan Provincial Center for Disease Control and Prevention. Permission was obtained from LPM managers before their participation in this study.

## Results

### Influenza prevalence

During the period from February to April of 2014, a total of 3943 environmental samples were collected from 380 LPMs covering all 122 counties/districts of Hunan province. The samples were tested for influenza A, H9, H7 and H5 using real-time RT-PCR assays. 1554 samples were positive for influenza A with a positive rate of 39.4%, of which 618 were positive for H9 subtype. H9 subtype counted for a majority, followed by H5 and H7 of these three subtypes of AIVs. Of the 618 samples positive for H9 subtype, 37 samples showed mix of H9 and H7 subtypes, 189 samples showed mix of H9 and H5 subtypes, and 39 samples showed mix of H9, H7 and H5 subtypes (Table 1).

**Table 1:** Results of real-time RT-PCR testing of environmental samples from live poultry markets in Hunan province, China.

Year/Month	Number of samples	Number of samples positive for							
		Influenza A (%)	H9^a^ (%)	H7^a^ (%)	H5^a^ (%)	H9, H7 (%)	H9, H5 (%)	H7, H5 (%)	H9, H7, H5 (%)
2014/02	1369	567 (41.4)	166 (12.1)	81 (5.9)	279 (20.4)	11 (0.8)	49 (3.6)	14 (1.0)	13 (0.9)
2014/03	1251	457 (36.5)	190 (15.2)	46 (3.7)	229 (18.3)	13 (1.0)	60 (4.8)	8 (0.6)	13 (1.0)
2014/04	1323	530 (40.1)	262 (19.8)	36 (2.7)	249 (18.8)	13 (1.0)	80 (6.0)	5 (0.4)	13 (1.0)
Total	3943	1554 (39.4)	618 (15.7)	163 (4.1)	757 (19.2)	37 (0.9)	189 (4.8)	27 (0.7)	39 (1.0)

^a^Including mixed with other subtypes.

The most heavily contaminated sample types with H9 subtype AIVs were sewage (18.0%) and poultry drinking water (22.2%) (Table 2). Poultry feces and poultry cages were moderately contaminated, with positive rates of 9.0% and 10.8% respectively. There was no statistically significant difference of H9 positive rates between poultry feces, poultry cage, chopping board and others. However, the H9 positive rates of sewage and poultry drinking water was higher than poultry feces, poultry cage, chopping board and others (S2 Table).

**Table 2:** Comparison of positive rates for H9 subtype of different environmental sample types detected by real-time RT-PCR.

Sample type	Number of samples	Number of samples positive for H9 (%)
Sewage	1147	207 (18.0)
Poultry drinking water	1191	264 (22.2)
Poultry feces	830	75 (9.0)
Poultry cage	491	53 (10.8)
Chopping board	223	14 (6.3)
Others^a^	61	5 (8.2)
Total	3943	618 (15.7)

^a^Others including poultry meat, feather, blood and eggs

The H9 AIV prevalence among different areas varied. The Huarong County located in the north of Hunan province had the highest positive rate of 76.5% in all 122 counties/districts of Hunan province. 98 (80.3%) of 122 counties/districts were contaminated with H9 subtype AIVs and no H9 AIV was detected in only 24 (19.7%) counties/districts. Areas with relatively high positive rates were mainly in the north, northeast, south and southwest of Hunan province (S3 Table). Of 380 LPMs involved, 200 (52.6%) markets were contaminated with H9 subtype AIVs.

### Phylogenetic analysis

Eleven H9N2 viruses were isolated from sewage and poultry drinking water in LPMs covering nine of fourteen cities of the province (Table 3). To better understand the evolutionary relationship of AIVs in LPMs in Hunan province, we sequenced the entire coding sequences of the genomes of the eleven isolates. The HA genes revealed a close relation between the eleven viruses, with 97.9%-99.9% nucleotide identities, while the NA genes shared 94.3%-99.9% nucleotide identities. The other six internal genes shared nucleotide identities of 95.6%-99.9%, 95.5%-99.9%, 97.2%-100%, 97.2%-99.9%, 97.0%-100%, 95.4%-99.8%, with the PB2, PB1, PA, NP, M and NS genes respectively of the eleven viruses.

**Table 3:** H9N2 avian influenza viruses isolated from live poultry markets with county/district, city of collection, date of collection and sample type.

Virus name	County/district, city of collection	Date of collection	Sample type
A/Environment/Hunan/18556/2014	Liuyang, Changsha	2/25/2014	Poultry drinking water
A/Environment/Hunan/27408/2014	Liuyang, Changsha	4/14/2014	Poultry drinking water
A/Environment/Hunan/27420/2014	Yuelu, Changsha	4/15/2014	Sewage
A/Environment/Hunan/28034/2014	Changsha, Changsha	4/21/2014	Poultry drinking water
A/Environment/Hunan/18355/2014	Fenghuang, Xiangxi	3/5/2014	Sewage
A/Environment/Hunan/18498/2014	Shifeng, Zhuzhou	3/10/2014	Poultry drinking water
A/Environment/Hunan/21403/2014	Xinhuang, Huaihua	3/11/2014	Cage swabs
A/Environment/Hunan/18489/2014	Zhenxiang, Hengyang	3/11/2014	Chopping board swabs
A/Environment/Hunan/18370/2014	Wuling, Changde	3/16/2014	Poultry drinking water
A/Environment/Hunan/25998/2014	Xinning, Shaoyang	3/21/2014	Poultry drinking water
A/Environment/Hunan/28028/2014	Linxiang, Yueyang	4/30/2014	Poultry drinking water

**Fig 1:**
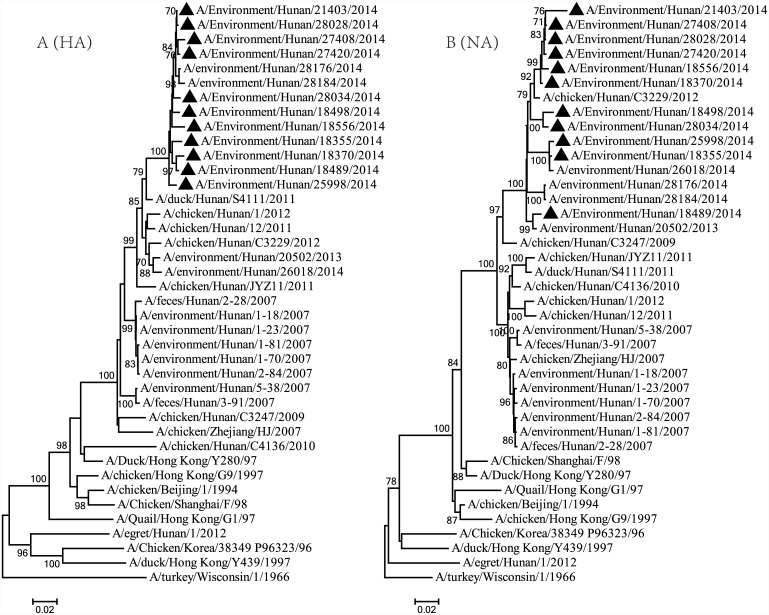
Phylogenetic analysis of the HA and NA genes of the H9N2 avian influenza viruses from live poultry markets in Hunan province, China. Phylogenetic trees of the entire coding nucleotide sequences for the genes of the H9N2 avian influenza viruses in this study compared with nucleotide sequences of representative H9N2 viruses of G1, Y280, and Y439 lineages. Bootstrap values over 70% are shown in the nodes. Solid triangles show the H9N2 viruses isolated from the live poultry markets in this study.

The phylogenetic relationships of all eight genes of the eleven H9N2 viruses were analyzed compared to H9N2 representative viruses and other H9N2 viruses isolated from Hunan province. Phylogenetically, the viruses also showed a high degree of homology despite being isolated from different cities of the province. Eight gene segments of all eleven viruses were in a large branch with the A/Chicken/Zhejiang/HJ/2007, which was thought as an ancestor of the novel H7N9 viruses (Fig 1, S1 Fig, S2 Fig and S3 Fig). All eleven viruses had the same gene constellations of genotype G57 or genotype S, which had PB2 and M genes of G1 lineage and other six genes of F98 lineage [10, 11]. No gene reassortment was found. In addition, six internal gene segments clustered with the H7N9 isolate A/Changsha/1/2013 of Hunan province. Although all these viruses belonged to one genotype, they still had some diversity among the individual gene segments. In the NA gene, A/Environment/Hunan/21403/2014 and A/Environment/Hunan/25998/2014 had a homology of less than 95% (94.3%) even though they were in the same branch. In the NS gene, A/Environment/Hunan/18556/2014, A/Environment/Hunan/18498/2014 and A/Environment/Hunan/28034/2014 were in a sub-branch, different from other strains.

### Molecular characteristics

The molecular characteristics of deduced amino acid sequences of the eleven H9N2 viruses were compared with the representative H9N2 strains circulating in Eurasia (Table 4). The analysis showed that the isolates had typical contemporary poultry H9N2 virus features. All the viruses except the A/Environment/Hunan/27408/2014 had a R-S-S-R connecting peptide in the cleavage site of the HA molecule, which is a typical feature of land avian influenza viruses [28]. The HA receptor binding sites had a 226L motif (H3 numbering), suggesting that these viruses could preferentially have affinity for human-like α-2,6-linked sialic acid receptors [29]. All the viruses had a 3-amino acid deletion at positions 63–65 in the NA stalk region. It has been reported that this deletion might be necessary for virus adaptation from wild birds to poultry [30]. No H274Y substitution was observed in the NA protein, which indicated that these viruses would be sensitive to NA inhibitors such as oseltamivir [31]. The S31N/G sequence of M2 protein suggested that all these viruses were resistant to amantadine and rimantadine [32]. All the viruses maintained 627E and 701D in PB2 of the low pathogenic AIV signatures [33–35].

**Table 4:** Comparison of critical amino acid residues in proteins of H9N2 avian influenza viruses from live poultry markets in Hunan province, China.

Virus Name	HA^a^							NA	M1	M2			NS1	PB1	PA	
	Receptor Binding Site						Cleavage Site	Stalk deletions								
	158	183	190	226	227	228		63–65	15	28	31	55	227	13	356	409
A/Environment/Hunan/18556/2014	N	N	T	L	M	G	RSSR	Yes	I	V	N	F	K	P	R	N
A/Environment/Hunan/27408/2014	N	N	T	L	M	G	RCSR	Yes	I	V	N	F	K	P	R	N
A/Environment/Hunan/27420/2014	N	N	T	L	M	G	RSSR	Yes	I	V	N	F	K	P	R	N
A/Environment/Hunan/28034/2014	N	N	T	L	M	G	RSSR	Yes	I	I	N	F	K	P	R	N
A/Environment/Hunan/18355/2014	N	N	T	L	M	G	RSSR	Yes	I	V	N	F	K	P	R	N
A/Environment/Hunan/18498/2014	N	N	T	L	M	G	RSSR	Yes	I	V	N	F	K	P	R	N
A/Environment/Hunan/21403/2014	N	N	T	L	M	G	RSSR	Yes	I	V	N	F	K	P	R	N
A/Environment/Hunan/18489/2014	N	N	T	L	M	G	RSSR	Yes	I	V	N	F	K	P	R	N
A/Environment/Hunan/18370/2014	N	N	T	L	M	G	RSSR	Yes	I	V	N	F	K	P	R	N
A/Environment/Hunan/25998/2014	N	N	T	L	M	G	RSSR	Yes	I	V	N	F	K	P	R	N
A/Environment/Hunan/28028/2014	N	N	T	L	M	G	RSSR	Yes	I	V	N	F	K	P	R	N

^a^According to the H3 numbering.

## Discussion

In this study, we collected environmental samples from LPMs covering all counties/districts of Hunan province, and tested the samples by real-time RT-PCR, virus isolation and whole-genome sequencing. We discovered that 15.7% samples were positive for H9, while more than half of the LPMs and more than four-fifths counties/districts were contaminated with H9 subtype viruses. The sequenced H9N2 viruses were highly homologous in gene sequence, belonged to the same genotype and had some specific molecular features. These results provide baseline information for the prevalence of H9N2 AIVs in LPMs of Hunan province, and provide a scientific basis for prevention and control of avian influenza in Hunan province and help early warning forecast for influenza pandemics.

Our findings provide further evidence that LPMs are heavily contaminated with AIVs [18, 36]. The positive rate for influenza A of Hunan province was 39.4%, higher than that of Nanchang, nearby Jiangxi province (~30%)[37]. The H9 subtype positive rate was also higher than that of nearby Guangdong province (11.8%)[38]. These results indicated that identification the first human case of H9N2 viruses of Hunan province was no accident. In various mixtures of subtypes of AIVs, the H9 subtype occupied a high proportion and was a predominant strain. That mixture of different HA subtype AIVs provided the possibility for the emergence of novel reassortant viruses and partially explained why the six internal gene segments of H7N9 and H10N8 viruses were derived from H9N2 viruses. Sewage and poultry drinking water were main issues for sampling and disinfection since they had the highest positive rates among different sample types. Geographical distribution of H9 subtype AIVs were mainly in the north, northeast, south and southwest of Hunan province. However little is known about why these viruses have this distribution pattern and how they spread.

Dongting lake wetland in Hunan province, which is on the East Asian-Australasian Flyway of migratory birds, plays an important role in the ecology of AIVs by supporting complex reassortment [39]. In this region H9N2 viruses isolated from wild birds and poultry are distinct [40]. Another aim of this study was to see whether reassortment of AIVs occurs in LPMs between poultry and wild birds. Our data demonstrated that all eleven viruses belonged to a same genotype and no gene reassortment was found.

Genotype G57 of H9N2 viruses became the single predominant H9N2 genotype in the farm chicken population in China, caused widespread outbreaks in 2010–2013 and provided all of their internal genes to the novel H7N9 viruses [10]. A previous study showed that two H9N2 viruses isolated from poultry markets in Hunan province in 2011 and 2012 exhibited genetic diversity, with their PB1 genes in different evolutional lineages [40]. However, the data from our study suggested all sequenced H9N2 viruses had a similar genome constellation, as well as belonged to genotype G57, which was still predominant in LPMs of Hunan province in 2014. Although the geographic distribution of our isolates covered eight of fourteen cities, we still did not found genotype diversity. One possible reason is circulation of live poultry trading leading to the spread of a single genotype of virus. Another possibility is that the range and numbers of our monitoring were not enough to find genetic reassortment. Therefore, we need to continue to strengthen avian influenza surveillance in LPMs and improve monitoring sensitivity.

Studies indicated that H9N2 viruses with 226L in the HA molecule could replicate in ferrets and be transmitted by direct contact [41]. This variant was found in 76% of the H9N2 viruses in chicken in Central Asia and the Middle East [42, 43]. Our findings demonstrated that all eleven viruses were 226L variants and several viruses had mutations in other key sites. The transmission and virulence of these viruses should be further tested and verified in mammalian animal models.

In summary, we conducted LPM surveys for AIVs in all 122 counties/districts of Hunan province in China and performed preliminary analysis of genetic characteristics of the H9N2 viruses isolated from LPMs of different cities. Our findings extend our knowledge of the ecology of H9N2 AIVs circulating in LPMs and provide significant insights into molecular evolution of these viruses. We emphasize the importance for continued surveillance of AIVs in LPMs for identification of further AIV evolution and response to potential influenza pandemics.

## Supporting Information

S1 Fig Phylogenetic analysis of the PB2 and PB1 genes of the H9N2 avian influenza viruses from live poultry markets in Hunan province, China. Phylogenetic trees of the entire coding nucleotide sequences for the genes of the H9N2 avian influenza viruses in this study compared with nucleotide sequences of representative H9N2 viruses of G1, Y280, and Y439 lineages. Bootstrap values over 70% are shown in the nodes. Solid triangles show the H9N2 viruses isolated from the live poultry markets in this study.
(EPS)

S2 Fig Phylogenetic analysis of the PA and NP genes of the H9N2 avian influenza viruses from live poultry markets in Hunan province, China. Phylogenetic trees of the entire coding nucleotide sequences for the genes of the H9N2 avian influenza viruses in this study compared with nucleotide sequences of representative H9N2 viruses of G1, Y280, and Y439 lineages. Bootstrap values over 70% are shown in the nodes. Solid triangles show the H9N2 viruses isolated from the live poultry markets in this study.
(EPS)

S3 Fig Phylogenetic analysis of the M and NS genes of the H9N2 avian influenza viruses from live poultry markets in Hunan province, China. Phylogenetic trees of the entire coding nucleotide sequences for the genes of the H9N2 avian influenza viruses in this study compared with nucleotide sequences of representative H9N2 viruses of G1, Y280, and Y439 lineages. Bootstrap values over 70% are shown in the nodes. Solid triangles show the H9N2 viruses isolated from the live poultry markets in this study.
(EPS)

S1 Table Geographical coordinates of 122 counties and names of live poultry markets for environmental surveillance of live poultry markets in Hunan province. (DOCX)

S2 Table P values of pairwise comparisons between different environmental sample types. (DOCX)

S3 Table Positive rates for H9 subtype of 122 counties in Hunan province. (DOCX)

S4 Table Information and real-time RT-PCR results of environmental samples from live poultry markets in Hunan province. (XLSX)
