# Supplementary material for: Detection and Genetic Characteristics of H9N2 Avian Influenza Viruses from Live Poultry Markets in Hunan Province, China
Source: PLoS One. 2015 Nov 10;10(11):e0142584. doi: 10.1371/journal.pone.0142584 (PMC4640513; doi:10.1371/journal.pone.0142584)
Supplement: S1 Table — (DOCX) [file pone.0142584.s004.docx]

S1 Table Geographical coordinates of 122 counties and names of live poultry markets for environmental surveillance of live poultry markets in Hunan province.

| **County/District name** | **Latitude** | **Longitude** | **Market name** | **Number of samples** |
| --- | --- | --- | --- | --- |
| Anhua County | 28.374089 | 111.212808 | da ma tou nong mao shi chang | 8 |
|  |  |  | liu xi nong mao shi chang | 3 |
|  |  |  | mei cheng nong mao shi chang | 10 |
|  |  |  | xiang zi shi chang | 9 |
| Anren County | 26.709053 | 113.269440 | an ping zhen nong mao shi chang | 3 |
|  |  |  | hong tai yang nong mao shi chang | 6 |
| Anxiang County | 29.451631 | 112.149135 | an xiang xian nong mao shi chang | 18 |
| Baojing County | 28.708882 | 109.650432 | bao jing xian huo qin shi chang | 6 |
|  |  |  | qian ling zhen shang mao zhong xin | 37 |
| Beihu District | 25.784058 | 113.011036 | dong feng nong mao shi chang | 6 |
|  |  |  | qi xing da shi chang | 8 |
|  |  |  | qi zuo qin lei pi fa shi chang | 85 |
|  |  |  | tong xin nong mao shi chang | 4 |
|  |  |  | zuo sha yuan nong mao shi chang | 3 |
|  |  |  | zuo yang wen shi rou ji jing xiao shang | 8 |
| Beita District | 27.246494 | 111.452200 | bei ta qu xi hu qiao nong mao shi chang | 18 |
| Chaling County | 26.812284 | 113.617666 | cha ling xian zhong xin nong mao shi chang | 7 |
|  |  |  | cheng xi nong mao shi chang | 3 |
|  |  |  | xiang gan nong mao shi chang | 3 |
| Changning City County | 26.363563 | 112.412603 | chang ning shi zhong xin ji mao shi chang | 17 |
| Changsha County | 28.286507 | 113.228971 | chang sha xian jin jing shi chang | 4 |
|  |  |  | chang sha xian ju zuo nong mao shi chang | 10 |
|  |  |  | chang sha xian quan tang shi chang | 4 |
| Chengbu County | 26.355276 | 110.311897 | cheng bu xian hao you duo chao shi | 3 |
|  |  |  | cheng bu xian nong mao shi chang | 15 |
| Chenxi County | 28.006347 | 110.183923 | chen xi shi chang | 3 |
|  |  |  | chen xi xian zhong xin shi chang | 4 |
|  |  |  | chen zhou shi chang | 5 |
|  |  |  | lao cheng shi chang | 3 |
| Cili County | 29.429973 | 111.139669 | ci li cheng xi nong mao shi chang | 20 |
|  |  |  | ci li xian cheng xi shi chang | 10 |
| Daoxian County | 25.526439 | 111.600795 | dao xian da shi chang | 8 |
|  |  |  | dao xian hong xing shi chang | 12 |
|  |  |  | dao xian huo qin pi fa shi chang | 11 |
|  |  |  | dao xian nong mao zong he shi chang | 16 |
|  |  |  | dao xian tuo la ji zhan shi chang | 6 |
|  |  |  | dao xian zhen xing jie shi chang | 3 |
|  |  |  | dao xian zong he nong mao shi chang | 3 |
| Daxiang District | 27.233320 | 111.454132 | da xiang qu dai jia ping nong mao shi chang | 12 |
|  |  |  | da xiang qu nan men kou nong mao shi chang | 9 |
|  |  |  | da xiang qu ying chun nong mao shi chang | 15 |
|  |  |  | shao yang zhong nan jia qin pi fa shi chang | 30 |
| Dingcheng District | 28.808345 | 111.656928 | ding cheng qu chao yang lu nong mao shi chang | 6 |
|  |  |  | ding cheng qu wei chang cai shi chang | 12 |
| Dongan County | 26.397549 | 111.299808 | dong an xian da shi chang | 18 |
|  |  |  | dong an xian lu jia wan shi chang | 18 |
| Dongkou County | 27.060320 | 110.575848 | dong kou xian da qiao nong mao shi chang | 3 |
|  |  |  | dong kou xian da zheng jie jia qin tu zai | 3 |
|  |  |  | dong kou xian sha ji ya xiang | 6 |
|  |  |  | dong kou xian wen chang shi chang | 3 |
|  |  |  | dong kou zhen da zheng shi chang | 3 |
| Furong District | 28.196244 | 113.041763 | zuo rong qu huo ju er pian nong mao shi | 3 |
|  |  |  | zuo rong qu huo ju zhong xin nong mao shi | 3 |
|  |  |  | zuo rong qu huo xing nong mao shi chang | 7 |
|  |  |  | zuo rong qu yin gang nong mao shi chang | 3 |
| Guidong County | 26.077619 | 113.944616 | cheng guan zhen nong mao shi chang | 3 |
|  |  |  | gui dong xian nong mao shi chang | 6 |
| Guiyang County | 25.754116 | 112.734146 | gui yang xian er shi chang | 6 |
|  |  |  | gui yang xian yi shi chang | 6 |
| Guzhang County | 28.616935 | 109.950732 | gu yang zhen da shi chang | 18 |
| Hanshou County | 28.873962 | 112.007632 | han shou xian xi tao shi chang | 3 |
|  |  |  | han shou xian yin zuo shi chang | 15 |
| Hecheng District | 27.549429 | 109.965087 | he cheng qu zhong xin shi chang | 26 |
| Hengdong County | 27.110479 | 113.013064 | zhong xin cai shi chang | 19 |
| Hengnan County | 26.764378 | 112.894810 | che jiang zhen nong mao shi chang | 9 |
|  |  |  | san tang zhen nong mao shi chang | 15 |
| Hengshan County | 27.221519 | 112.805557 | kai yun shang mao shi chang | 18 |
| Hengyang County | 27.117106 | 112.367081 | heng yang xian cheng dong shi chang | 14 |
|  |  |  | heng yang xian cheng xi shi chang | 16 |
|  |  |  | heng yang xian jin lan cai shi chang | 9 |
|  |  |  | hong shi zhen nong mao shi chang | 20 |
|  |  |  | xi du zhen cheng xi shi chang | 6 |
| Heshan District | 28.579397 | 112.374162 | chang de lao xiang li tu ji ya | 5 |
|  |  |  | liu qiu sheng qin lei jia gong dian | 5 |
|  |  |  | nong jia xiang li | 5 |
|  |  |  | tao hua lun shi chang | 5 |
|  |  |  | tao hua lun shi chang shi chang | 5 |
|  |  |  | tuan zhou shi chang | 20 |
|  |  |  | yi shi jia qin | 5 |
| Hetang District | 27.855877 | 113.173492 | bai yun nong mao shi chang | 3 |
|  |  |  | cai shi chang | 6 |
|  |  |  | gui hua ban shi chu shi chang | 4 |
|  |  |  | he ye tang nong mao shi chang | 4 |
|  |  |  | si san da shi chang | 4 |
|  |  |  | zuo jia wan shi chang | 4 |
| Hongjiang City County | 27.203906 | 109.833841 | che zhan shi chang | 3 |
|  |  |  | qian cheng shi chang | 18 |
|  |  |  | tang zuo shi chang | 12 |
| Huarong County | 29.484987 | 112.674172 | hua rong xian nan men shi chang | 20 |
|  |  |  | hua rong xian qing nian lu shi chang | 3 |
|  |  |  | le le da cai shi | 11 |
| Huayuan County | 28.572029 | 109.482075 | hua yuan xian cheng guan shi chang | 3 |
|  |  |  | hua yuan xian hua yuan zhen shi chang | 6 |
|  |  |  | hua yuan xian jin san jiao shi chang | 21 |
|  |  |  | xi men kou nong mao shi chang | 6 |
| Huitong County | 26.872813 | 109.720963 | lin cheng zhen cheng dong shi chang | 18 |
| Jiahe County | 25.587524 | 112.369022 | bing xue shi chang | 11 |
|  |  |  | dang xiao shi chang | 6 |
|  |  |  | feng he zuo | 3 |
|  |  |  | hong fa shi chang | 15 |
|  |  |  | jia he da shi chang | 6 |
|  |  |  | zhu er shi chang | 3 |
|  |  |  | zhu quan shi chang | 14 |
| Jianghua County | 25.185593 | 111.579305 | jiang hua xian nong mao shi chang | 24 |
| Jiangyong County | 25.273543 | 111.343914 | jiang yong cheng bei nong mao shi chang | 13 |
|  |  |  | jiang yong shang zuo shi chang | 3 |
|  |  |  | jiang yong xian tao chuan shi chang | 3 |
| Jingzhou County | 26.575075 | 109.696244 | fei shan shi chang | 3 |
|  |  |  | yu lin zuo shi chang | 12 |
|  |  |  | zuo shan kou | 6 |
| Jinshi City County | 29.509976 | 111.905000 | jin shi shi he ping shi chang | 6 |
|  |  |  | jin shi shi san yan qiao nong mao shi chang | 6 |
|  |  |  | jin shi shi san zhou zuo shi chang | 8 |
|  |  |  | jin shi shi xiang yang jie nong mao shi chang | 3 |
| Jishou City County | 28.262356 | 109.698089 | hong qi men nong mao shi chang | 18 |
|  |  |  | qian zhou wei ye shi chang | 18 |
|  |  |  | shi jia chong nong mao shi chang | 18 |
| Junshan District | 29.461169 | 113.006433 | gua kou shi chang | 9 |
|  |  |  | jun liu shi chang | 15 |
|  |  |  | jun shan gua kou shi chang | 6 |
|  |  |  | jun shan jun liu shi chang | 6 |
| Kaifu District | 28.291609 | 112.987883 | kai fu qu mao jia qiao qin xu shi chang | 35 |
|  |  |  | kai fu qu sha hu qiao shi chang | 6 |
| Lanshan County | 25.369903 | 112.196728 | lan shan ta feng zhen er shi chang | 6 |
|  |  |  | lan shan tai ping shi chang | 6 |
|  |  |  | lan shan xian liang shi ju | 4 |
|  |  |  | lan shan xian tai ping shi chang | 6 |
|  |  |  | lan shan xian yi shi chang | 34 |
| Leiyang City County | 26.410936 | 112.927759 | zuo yang shi jin shan shi chang | 24 |
|  |  |  | zuo yang shi lao nong mao shi chang | 12 |
| Lengshuitan District | 26.461074 | 111.592147 | feng huang yuan zhong xin shi chang | 3 |
|  |  |  | leng shui tan huang ni jing shi chang | 87 |
|  |  |  | leng shui tan mei wan cai shi chang | 3 |
|  |  |  | leng shui tan qu hong ping nong mao da shi | 3 |
| Li County | 29.681789 | 111.647079 | zuo xian hong wei shi chang | 18 |
| Lianyuan City County | 27.692544 | 111.664320 | lian yuan lan tian huo qin jiao yi shi chang | 14 |
|  |  |  | xin hua cheng nan shi chang | 1 |
| Liling City County | 27.670321 | 113.468535 | tai yi shi chang | 18 |
| Lingling District | 26.221887 | 111.631136 | ling ling qu an zhi shi chang | 27 |
|  |  |  | ling ling xu jia jing huo qin pi fa bu | 9 |
| Linli County | 29.516615 | 111.637477 | lin zuo xian chao yang shi chang | 12 |
|  |  |  | lin zuo xian yi shi chang | 6 |
| Linwu County | 25.275561 | 112.563461 | cheng guan zhen xin shi chang | 9 |
| Linxiang City County | 29.522786 | 113.524432 | bei zheng jie cai shi chang | 16 |
|  |  |  | san jiao ping cai shi chang | 10 |
|  |  |  | wu li cai shi chang | 10 |
| Liuyang City County | 28.196329 | 113.720169 | zuo yang ji li nong mao shi chang | 11 |
|  |  |  | zuo yang shi de sheng qin xu pi fa shi | 6 |
|  |  |  | zuo yang shi gong jia qiao nong mao shi chang | 6 |
|  |  |  | zuo yang shi jie fang lu sheng xian shi chang | 5 |
|  |  |  | zuo yang shi shu cai pi fa shi chang | 4 |
| Longhui County | 27.113974 | 111.032435 | long hui xian chao yang shi chang | 18 |
|  |  |  | long hui xian huo qin jiao yi shi chang | 21 |
|  |  |  | long hui xian zhong xin shi chang | 15 |
| Longshan County | 29.457648 | 109.443795 | long shan xian cheng bei shi chang | 18 |
|  |  |  | long shan xian cheng nan shi chang | 18 |
| Louxing District | 27.729920 | 112.001935 | lou xing qu jin gu shi chang | 20 |
|  |  |  | lou xing qu zhong xing shi chang | 10 |
| Lusong District | 27.824408 | 113.159727 | he jia tu nong mao shi chang | 5 |
|  |  |  | heng feng jia qin xing | 3 |
|  |  |  | man jiang hong nong mao shi chang | 3 |
|  |  |  | qi ji nong mao shi chang | 3 |
|  |  |  | zhong xin nong mao shi chang | 3 |
| Luxi County | 28.216637 | 110.219607 | bai sha da shi chang | 18 |
|  |  |  | wu xi da shi chang | 18 |
| Mayang County | 27.865561 | 109.802578 | ma yang cheng bei shi chang | 14 |
|  |  |  | ma yang cheng nan shi chang | 5 |
| Miluo City County | 28.743035 | 113.161454 | cheng xi ji mao shi chang | 9 |
|  |  |  | da zhong lu ji mao shi chang | 12 |
|  |  |  | zhong xin ji mao shi chang | 29 |
| Nan County | 29.371237 | 112.409331 | bao lin xiang ji mao shi chang | 15 |
|  |  |  | xiang e bian cai shi chang | 10 |
|  |  |  | xing sheng lu ji mao shi chang | 30 |
| Nanyue District | 27.243947 | 112.729951 | cheng zhong huo qin shi chang | 6 |
|  |  |  | nan yue nong mao shi chang | 6 |
|  |  |  | nan yue zhong xin shi chang | 6 |
| Ningxiang County | 28.182182 | 112.339754 | ning xiang shan mu qiao nong mao shi chang | 10 |
|  |  |  | ning xiang xian huang cai shi chang | 3 |
|  |  |  | ning xiang xian hui tang zhen cai shi chang | 3 |
| Ningyuan County | 25.570976 | 111.945802 | he ting zhen lu bian tan dian | 3 |
|  |  |  | ning yuan xian gao feng shi chang | 6 |
|  |  |  | ning yuan xian huo qin pi fa shi chang | 6 |
|  |  |  | ning yuan xian leng jiang shi chang | 6 |
|  |  |  | ning yuan xian tong shan nong mao shi chang | 11 |
| Phoenix County | 28.076969 | 109.567273 | tuan jie qiao shi chang | 16 |
|  |  |  | zuo jiang zhen shi chang | 20 |
| Pingjiang County | 28.762976 | 113.653404 | cheng dong cai shi chang | 6 |
|  |  |  | lian yun cai shi chang | 3 |
|  |  |  | ping jiang dong jie cai shi chang | 6 |
|  |  |  | ping jiang xian wu shi zhen zong he nong mao | 3 |
|  |  |  | tian yue nong mao shi chang | 4 |
|  |  |  | tong jia ling cai shi chang | 5 |
|  |  |  | yue chi tang cai shi chang | 3 |
| Qidong County | 26.765872 | 111.942176 | qi dong cheng dong shi chang | 4 |
|  |  |  | qi dong xian cheng zhong shi chang | 20 |
| Qiyang County | 26.580122 | 111.840659 | qi yang huang fu ping shi chang | 25 |
|  |  |  | qi yang lu jia dian shi chang | 8 |
|  |  |  | qi yang wang zuo yuan shi chang | 14 |
|  |  |  | qi yang xi bei nong mao shi chang | 28 |
| Quyuan Administration Area | 28.743035 | 113.161454 | fu min jie shi chang | 16 |
|  |  |  | qu yuan ji mao shi chang | 3 |
|  |  |  | zuo fang shi chang | 19 |
| Rucheng County | 25.550505 | 113.684979 | cheng guan zhen nong mao shi chang | 6 |
|  |  |  | ru cheng yi zhong pang | 3 |
|  |  |  | xiao man jia qin dian | 3 |
| Sangzhi County | 29.399721 | 110.163925 | sang zhi xian zuo yuan shi chang | 30 |
| Shaodong County | 27.258941 | 111.744261 | shao dong xian bai bao cai shi chang | 3 |
|  |  |  | shao dong xian cheng lan nong mao shi chang | 3 |
|  |  |  | shao dong xian he hua nong mao shi chang | 6 |
|  |  |  | shao dong xian sheng li jie nong mao shi chang | 3 |
| Shaoshan City County | 27.936380 | 112.523550 | shao shan zhong xin nong mao shi chang | 22 |
| Shaoyang County | 26.990638 | 111.273801 | shao yang xian bai cang nong mao shi chang | 4 |
|  |  |  | shao yang xian tang du kou da ling shi chang | 3 |
|  |  |  | shao yang xian tang du kou shi wan shi chang | 3 |
|  |  |  | shao yang xian tang du kou zhen er shi chang | 16 |
|  |  |  | shao yang xian tang du kou zhen yi shi chang | 20 |
|  |  |  | shao yang xian zha tan huo qin shi chang | 15 |
| Shifeng District | 27.866765 | 113.117691 | ai hua jia qin pi fa bu | 6 |
|  |  |  | bing ji han jiang tu ji | 4 |
|  |  |  | jiu yuan shi chang | 3 |
|  |  |  | li min pi fa dian | 4 |
|  |  |  | ma wu jia qin pi fa | 5 |
|  |  |  | tong luo wan nong mao shi chang | 3 |
|  |  |  | wen shi tu ji te yue jing xiao dian | 4 |
|  |  |  | xiang dong nong mao shi chang | 3 |
|  |  |  | xiao li jia qin pi fa | 4 |
|  |  |  | zuo zuo leng ku | 4 |
| Shigu District | 26.902381 | 112.610689 | hou zai men shi chang | 25 |
|  |  |  | sang yuan nong chan pin pi fa shi chang | 5 |
|  |  |  | shi gu qu yan qi qiao shi chang | 25 |
| Shimenn County | 29.698007 | 111.024849 | shi men xian chu jiang shi chang | 3 |
|  |  |  | shi men xian zhan xi lu shi chang | 15 |
| Shuangfeng County | 27.459140 | 112.192447 | shuang feng xian bai yan shi chang | 4 |
|  |  |  | shuang feng xian cheng bei nong mao shi chang | 7 |
|  |  |  | shuang feng xian yong feng nong mao shi chang | 8 |
| Shuangpai County | 25.961913 | 111.659964 | shuang pai xian da shi chang | 15 |
|  |  |  | shuang pai xian nong mao shi chang | 12 |
| Shuangqing District | 27.232910 | 111.497154 | shuang qing qu san yan jing nong mao shi chang | 23 |
| Suining County | 26.581955 | 110.155653 | sui ning xian lao jie nong mao shi chang | 15 |
|  |  |  | sui ning xian lv zhou nong mao shi chang | 16 |
|  |  |  | sui ning xian zhong xin nong mao shi chang | 29 |
| Suxian District | 25.800365 | 113.042439 | dong yuan ge nong mao shi chang | 10 |
|  |  |  | su xian qiao cai shi chang | 16 |
|  |  |  | su yuan nong mao shi chang | 11 |
| Taojiang County | 28.518082 | 112.155819 | tao hua jiang zhen shi chang | 5 |
|  |  |  | zhang jia ma tou nong mao shi chang | 25 |
|  |  |  | zhen hua shi chang | 20 |
| Taoyuan County | 28.918908 | 111.288639 | tao yuan xian dong jie cai shi chang | 3 |
|  |  |  | tao yuan xian hao xing nong mao shi chang | 15 |
| Tianxin District | 28.114704 | 112.969226 | qing yuan nong mao shi chang | 5 |
|  |  |  | tian xin qu xin tian nong mao shi chang | 12 |
|  |  |  | tian xin qu xin yao lu kou cai shi chang | 6 |
|  |  |  | tie dao nong mao shi chang | 3 |
| Tianyuan District | 27.830756 | 113.123485 | tian yuan qu nong mao shi chang | 16 |
|  |  |  | yao hua nong mao shi chang | 3 |
| Tongdaoxian County | 26.158038 | 109.784360 | tong dao cheng dong shi chang | 15 |
|  |  |  | tong dao jia qin shi chang | 6 |
|  |  |  | tong dao xian xi shi chang | 3 |
|  |  |  | tong dao zhong xin shi chang | 3 |
| Wangcheng County | 28.267157 | 112.826800 | gao tang ling jie dao hong wang da shi chang | 5 |
|  |  |  | wang cheng qu qiao kou shang mao cheng | 3 |
|  |  |  | wang cheng qu tong guan jia yuan cai shi chang | 5 |
| Wugang City County | 26.726600 | 110.631884 | wu gang shi gong ye pin nong mao shi chang | 6 |
|  |  |  | wu gang shi nong mao shi chang | 15 |
|  |  |  | wu gang shi san pai lu shi chang | 6 |
|  |  |  | wu gang shi tang chang pi fa shi chang | 9 |
| Wuling District | 29.172033 | 111.866323 | wu ling qu chang sheng qiao shi chang | 5 |
|  |  |  | wu ling qu dan yang lou shi chang | 3 |
|  |  |  | wu ling qu gan lu si shi chang | 3 |
|  |  |  | wu ling qu huo che zhan shi chang | 8 |
|  |  |  | wu ling qu long gang xiang nong mao shi chang | 6 |
|  |  |  | wu ling qu mei jiang shi chang | 65 |
|  |  |  | wu ling qu qing yang ge nong mao shi chang | 19 |
|  |  |  | wu ling qu san zuo shi chang | 7 |
|  |  |  | wu ling qu zi lan shi chang | 3 |
|  |  |  | wu ling qu zi qiao shi chang | 4 |
| Wulingyuan District | 29.345727 | 110.550431 | wu ling yuan qu hong yuan shi chang | 20 |
|  |  |  | wu ling yuan qu nong mao shi chang | 10 |
| Xiangtan County | 27.709680 | 112.738599 | hua shi zong he nong mao shi chang | 3 |
|  |  |  | jin peng nong mao shi chang | 3 |
|  |  |  | jin shi nong mao shi chang | 3 |
|  |  |  | she fu nong mao shi chang | 6 |
|  |  |  | shi tan nong mao shi chang | 3 |
|  |  |  | yi su he nong mao shi chang | 3 |
|  |  |  | yun hu qiao nong mao shi chang | 3 |
| Xiangxiang City County | 27.778380 | 112.327749 | chang cai shi chang | 3 |
|  |  |  | dong feng cai shi chang | 9 |
|  |  |  | gong mao xin qu nong mao shi chang | 3 |
|  |  |  | hu tie cai shi chang | 4 |
|  |  |  | nan men cai shi chang | 13 |
|  |  |  | xin xiang lu hu tie shi chang | 3 |
|  |  |  | zhong xin nong mao shi chang | 8 |
| Xiangyin County | 28.768741 | 112.777845 | gao ling shi chang | 6 |
|  |  |  | qiao dong shi chang | 9 |
|  |  |  | tong da shi chang | 6 |
|  |  |  | xiang yin jian xin shi chang | 17 |
| Xinhua County | 27.726520 | 111.327413 | xin hua cheng nan shi chang | 7 |
|  |  |  | xin hua xian shang mei zhen li xin qiao nong | 3 |
|  |  |  | xin hua xian tian hua nan lu nong mao shi | 3 |
| Xinhuang County | 27.358028 | 109.171883 | xin huang shi chang | 18 |
| Xinning County | 26.433419 | 110.856621 | xin ning xian jin lai zhong xin shi chang | 23 |
| Xinshao County | 27.320916 | 111.458659 | xin shao xian jin san jiao shi chang | 18 |
| Xintian County | 25.906570 | 112.221576 | cheng guan wu xiang zuo shi chang | 4 |
|  |  |  | xin tian cheng dong xi men shi chang | 3 |
|  |  |  | xin tian tian jia xiang xiu feng shi chang | 5 |
|  |  |  | xin tian yong de li cai chang | 23 |
|  |  |  | xin zuo shi chang | 3 |
| Xupu County | 27.909988 | 110.594001 | zuo pu xian cheng bei shi chang | 12 |
|  |  |  | zuo pu xian cheng nan shi chang | 12 |
| Yanfeng District | 26.888737 | 112.616386 | yan feng qu xian ji xiang nong mao shi chang | 30 |
| Yanling County | 26.366879 | 113.844988 | yan ling xian nong mao shi chang | 12 |
| Yizhang County | 25.399960 | 112.948819 | cheng guan zhen huo qin pi fa shi chang | 12 |
|  |  |  | jiang jia wan shi chang | 6 |
|  |  |  | nan jing dong shi chang | 9 |
|  |  |  | si fang jing shi chang | 3 |
| Yongding District | 29.133870 | 110.474653 | yong ding qu da yong qiao shi chang | 10 |
|  |  |  | yong ding qu qi feng shi chang | 10 |
|  |  |  | yong ding qu xin feng shi chang | 10 |
| Yongshun County | 29.001441 | 109.851254 | yong shun xian huo qin shi chang | 18 |
| Yongxing County | 26.127151 | 113.116522 | cheng guan nong mao shi chang | 9 |
|  |  |  | cheng guan san fa shi chang | 3 |
|  |  |  | cheng guan shui nan shi chang | 6 |
| You County | 27.100288 | 113.517609 | hai kang nong mao shi chang | 6 |
|  |  |  | nan men nong mao shi chang | 3 |
|  |  |  | xi men xia nong mao shi chang | 3 |
|  |  |  | xiang dong nong mao shi chang | 6 |
| Yuanjiang City County | 28.847042 | 112.355955 | zuo jiang shi zhong xin cai shi chang | 10 |
|  |  |  | zuo jiang xiang bei shi chang | 40 |
| Yuanling County | 28.452723 | 110.393844 | zuo ling cheng xi shi chang | 3 |
|  |  |  | zuo ling tian ning shi chang | 18 |
| Yuelu District | 28.194050 | 112.918929 | wang yue hu sheng xian shi chang | 3 |
|  |  |  | yue lu qu ge lin xing cheng cai shi chang | 12 |
|  |  |  | yue lu qu xiang yi nong mao shi chang | 3 |
|  |  |  | yue lu qu yin pen ling sheng xian shi chang | 3 |
| Yuetang District | 27.904897 | 112.991424 | ban tang pu nong mao shi chang | 8 |
|  |  |  | chao yang cai shi chang | 4 |
|  |  |  | jiu zhou nong mao shi chang | 3 |
|  |  |  | mei ji nong mao shi chang | 3 |
|  |  |  | san zhong cai shi chang | 3 |
|  |  |  | zuo rong nong mao shi chang | 5 |
| Yueyang County | 29.220220 | 113.253400 | da tong ling shi chang | 9 |
|  |  |  | da yuan ling shi chang | 9 |
|  |  |  | da zhou ling shi chang | 3 |
|  |  |  | yue yang xian zhong xin shi chang | 15 |
| Yueyanglou District | 29.371844 | 113.129751 | ba zi men shi chang | 49 |
|  |  |  | feng qiao hu jia qin pi fa shi chang | 6 |
|  |  |  | hua ban qiao nong mao shi chang | 6 |
|  |  |  | hua rong xian nan men shi chang | 1 |
|  |  |  | le le da cai shi | 1 |
|  |  |  | ma hao qin dan pi fa shi chang | 12 |
|  |  |  | mei xi qiao shi chang | 19 |
|  |  |  | wu li pai nong mao shi chang | 6 |
| Yuhu District | 27.854074 | 112.897171 | ai guo qiao nong mao shi chang | 3 |
|  |  |  | jiang lu nong mao shi chang | 11 |
|  |  |  | jiang nan da shi chang | 3 |
|  |  |  | jiu hua shan shan nong mao shi chang | 3 |
|  |  |  | min zhu lu cai shi chang | 10 |
|  |  |  | nan pan ling nong mao shi chang | 3 |
|  |  |  | sha zi ling nong mao shi chang | 6 |
| Yuhua District | 28.117080 | 113.049327 | yang jia shan qin xu pi fa shi chang | 15 |
|  |  |  | yu hua qu shi ma jia qin shi chang | 15 |
| Yunxi District | 29.473574 | 113.271908 | yue hua wang jia ling shi chang | 9 |
|  |  |  | yun xi qu lu kou shi chang | 12 |
|  |  |  | yun xi qu si tong shi chang | 6 |
|  |  |  | yun xi shi chang | 5 |
|  |  |  | yun xi si tong shi chang | 3 |
| Zhengxiang District | 26.896507 | 112.603297 | gao xin qu san tai cai shi chang | 10 |
|  |  |  | heng xi shi chang | 20 |
|  |  |  | hua xin san tai cai shi chang | 10 |
|  |  |  | zheng xiang qu heng xi shi chang | 10 |
| Zhijiang County | 27.443411 | 109.685140 | zuo jiang huo qin shi chang | 5 |
|  |  |  | zuo jiang shi chang | 5 |
|  |  |  | zuo jiang zhong xin shi chang | 5 |
| Zhongfang County | 27.545301 | 110.202667 | zhong fang xian ya yan shi chang | 4 |
|  |  |  | zhong fang xian zhong xin shi chang | 9 |
|  |  |  | zhong fang zhen shi chang | 6 |
| Zhuhui District | 26.894660 | 112.620174 | mao ping qin lei pi fa shi chang | 5 |
|  |  |  | zhu zuo qu heng zhou shi chang | 40 |
|  |  |  | zhu zuo qu san hua pi fa shi chang | 15 |
|  |  |  | zhu zuo qu san tai cai shi chang | 25 |
| Zhuzhou County | 27.542523 | 113.165585 | lu kou zhen xiang yang nong mao shi chang | 21 |
| Zixing City County | 25.976246 | 113.236148 | zi xing shi xin qu nong mao shi chang | 16 |
| Ziyang District | 28.590956 | 112.324476 | san yi jie nong mao shi chang | 5 |
|  |  |  | zi yang qu xue men kou shi chang | 45 |
