# Supplementary material for: Detection and Genetic Characteristics of H9N2 Avian Influenza Viruses from Live Poultry Markets in Hunan Province, China
Source: PLoS One. 2015 Nov 10;10(11):e0142584. doi: 10.1371/journal.pone.0142584 (PMC4640513; doi:10.1371/journal.pone.0142584)
Supplement: S2 Table — (DOCX) [file pone.0142584.s005.docx]

S2 Table. P values of pairwise comparisons between different environmental sample types.

|  | Sewage | Poultry drinking water | Poultry feces | Poultry cage | Chopping board | Others |
| --- | --- | --- | --- | --- | --- | --- |
| Sewage | - |  |  |  |  |  |
| Poultry drinking water | 0.013 | - |  |  |  |  |
| Poultry feces | 0.000 | 0.000 | - |  |  |  |
| Poultry cage | 0.000 | 0.000 | 0.297 | - |  |  |
| Chopping board | 0.000 | 0.000 | 0.189 | 0.055 | - |  |
| Others | 0.049 | 0.010 | 0.825 | 0.533 | 0.595 | - |
