# Supplementary material for: Detection and Genetic Characteristics of H9N2 Avian Influenza Viruses from Live Poultry Markets in Hunan Province, China
Source: PLoS One. 2015 Nov 10;10(11):e0142584. doi: 10.1371/journal.pone.0142584 (PMC4640513; doi:10.1371/journal.pone.0142584)
Supplement: S3 Table — (DOCX) [file pone.0142584.s006.docx]

S3 Table Positive rates for H9 subtype of 122 counties in Hunan province.

| County name | Number of samples | Number of samples for H9 positive | Positive rate for H9 (%) |
| --- | --- | --- | --- |
| Huarong County | 34 | 26 | 76.47 |
| Pingjiang County | 30 | 20 | 66.67 |
| Linxiang City County | 36 | 22 | 61.11 |
| Xinhuang County | 18 | 11 | 61.11 |
| Shuangpai County | 27 | 15 | 55.56 |
| Jiangyong County | 19 | 10 | 52.63 |
| Yuhu District | 39 | 18 | 46.15 |
| Dongan County | 36 | 16 | 44.44 |
| Yueyanglou District | 100 | 42 | 42.00 |
| Hongjiang City County | 33 | 13 | 39.39 |
| Jianghua County | 24 | 9 | 37.50 |
| Xinning County | 23 | 8 | 34.78 |
| Hecheng District | 26 | 9 | 34.62 |
| Xiangyin County | 38 | 13 | 34.21 |
| Guidong County | 9 | 3 | 33.33 |
| Guiyang County | 12 | 4 | 33.33 |
| Linli County | 18 | 6 | 33.33 |
| Hetang District | 25 | 8 | 32.00 |
| Mayang County | 19 | 6 | 31.58 |
| Furong District | 16 | 5 | 31.25 |
| Shaoyang County | 61 | 19 | 31.15 |
| Yueyang County | 36 | 11 | 30.56 |
| Daxiang District | 66 | 20 | 30.30 |
| Yunxi District | 35 | 10 | 28.57 |
| Liuyang City County | 32 | 9 | 28.13 |
| Huitong County | 18 | 5 | 27.78 |
| Yuetang District | 26 | 7 | 26.92 |
| Sangzhi County | 30 | 8 | 26.67 |
| Shaodong County | 15 | 4 | 26.67 |
| Xintian County | 38 | 10 | 26.32 |
| Zhongfang County | 19 | 5 | 26.32 |
| Zhuzhou County | 21 | 5 | 23.81 |
| Quyuan Administration Area | 38 | 9 | 23.68 |
| Anxiang County | 18 | 4 | 22.22 |
| Dingcheng District | 18 | 4 | 22.22 |
| Junshan District | 36 | 8 | 22.22 |
| Linwu County | 9 | 2 | 22.22 |
| Xinshao County | 18 | 4 | 22.22 |
| Jinshi City County | 23 | 5 | 21.74 |
| Tianyuan District | 19 | 4 | 21.05 |
| Chenxi County | 15 | 3 | 20.00 |
| Wugang City County | 36 | 7 | 19.44 |
| Tianxin District | 26 | 5 | 19.23 |
| Lengshuitan District | 96 | 18 | 18.75 |
| Tongdaoxian County | 27 | 5 | 18.52 |
| Jiahe County | 58 | 10 | 17.24 |
| Hanshou County | 18 | 3 | 16.67 |
| Li County | 18 | 3 | 16.67 |
| Shimenn County | 18 | 3 | 16.67 |
| Xiangtan County | 24 | 4 | 16.67 |
| Nan County | 55 | 9 | 16.36 |
| Yuelu District | 21 | 3 | 14.29 |
| Shuangqing District | 23 | 3 | 13.04 |
| Miluo City County | 50 | 6 | 12.00 |
| Yuanjiang City County | 50 | 6 | 12.00 |
| Daoxian County | 59 | 7 | 11.86 |
| Suining County | 60 | 7 | 11.67 |
| Anren County | 9 | 1 | 11.11 |
| Beita District | 18 | 2 | 11.11 |
| Changsha County | 18 | 2 | 11.11 |
| Chengbu County | 18 | 2 | 11.11 |
| Jishou City County | 54 | 6 | 11.11 |
| Yongxing County | 18 | 2 | 11.11 |
| Jingzhou County | 21 | 2 | 9.52 |
| Ningyuan County | 32 | 3 | 9.38 |
| Qiyang County | 75 | 7 | 9.33 |
| Longhui County | 54 | 5 | 9.26 |
| Shaoshan City County | 22 | 2 | 9.09 |
| Wuling District | 123 | 11 | 8.94 |
| Beihu District | 114 | 10 | 8.77 |
| Lingling District | 36 | 3 | 8.33 |
| Xinhua County | 13 | 1 | 7.69 |
| Baojing County | 43 | 3 | 6.98 |
| Xiangxiang City County | 43 | 3 | 6.98 |
| Yizhang County | 30 | 2 | 6.67 |
| Zhijiang County | 15 | 1 | 6.67 |
| Ziyang District | 50 | 3 | 6.00 |
| Lusong District | 17 | 1 | 5.88 |
| Guzhang County | 18 | 1 | 5.56 |
| Hengshan County | 18 | 1 | 5.56 |
| Liling City County | 18 | 1 | 5.56 |
| Luxi County | 36 | 2 | 5.56 |
| Taoyuan County | 18 | 1 | 5.56 |
| Yongshun County | 18 | 1 | 5.56 |
| You County | 18 | 1 | 5.56 |
| Suxian District | 37 | 2 | 5.41 |
| Shifeng District | 40 | 2 | 5.00 |
| Qidong County | 24 | 1 | 4.17 |
| Heshan District | 50 | 2 | 4.00 |
| Taojiang County | 50 | 2 | 4.00 |
| Shigu District | 55 | 2 | 3.64 |
| Louxing District | 30 | 1 | 3.33 |
| Yuhua District | 30 | 1 | 3.33 |
| Longshan County | 36 | 1 | 2.78 |
| Kaifu District | 41 | 1 | 2.44 |
| Zhuhui District | 85 | 2 | 2.35 |
| Lanshan County | 56 | 1 | 1.79 |
| Hengyang County | 65 | 1 | 1.54 |
| Anhua County | 30 |  | 0.00 |
| Chaling County | 13 |  | 0.00 |
| Changning City County | 17 |  | 0.00 |
| Cili County | 30 |  | 0.00 |
| Dongkou County | 18 |  | 0.00 |
| Hengdong County | 19 |  | 0.00 |
| Hengnan County | 24 |  | 0.00 |
| Huayuan County | 36 |  | 0.00 |
| Leiyang City County | 36 |  | 0.00 |
| Lianyuan City County | 15 |  | 0.00 |
| Nanyue District | 18 |  | 0.00 |
| Ningxiang County | 16 |  | 0.00 |
| Phoenix County | 36 |  | 0.00 |
| Rucheng County | 12 |  | 0.00 |
| Shuangfeng County | 19 |  | 0.00 |
| Wangcheng County | 13 |  | 0.00 |
| Wulingyuan District | 30 |  | 0.00 |
| Xupu County | 24 |  | 0.00 |
| Yanfeng District | 30 |  | 0.00 |
| Yanling County | 12 |  | 0.00 |
| Yongding District | 30 |  | 0.00 |
| Yuanling County | 21 |  | 0.00 |
| Zhengxiang District | 50 |  | 0.00 |
| Zixing City County | 16 |  | 0.00 |
| Total | 3943 | 618 | 15.67 |
